# Posterior talar morphology as a risk factor for bony posterior ankle impingement syndrome: A propensity score-matched cohort study

**DOI:** 10.1016/j.asmart.2026.10.005

**Published:** 2026-10-27

**Authors:** Yuta Mori, Yasutaka Murahashi, Tomoaki Kamiya, Takashi Matsumura, Makoto Emori, Kota Watanabe, Atsushi Teramoto

**Affiliations:** a Department of Orthopaedic Surgery, Sapporo Medical University, Sapporo, Hokkaido, Japan; b Department of Orthopaedic Surgery, Obihiro Kyokai Hospital, Obihiro, Hokkaido, Japan; c Second Division of Physical Therapy, School of Health Sciences, Sapporo Medical University, Sapporo, Hokkaido, Japan; d Department of Sports Medicine, Sapporo Medical University, Sapporo, Hokkaido, Japan

**Keywords:** Ankle radiography, os trigonum, Posterior ankle impingement syndrome, Stieda's process

## Abstract

**Background:**

Bony posterior ankle impingement syndrome (PAIS) is associated with posterior talar morphology, including os trigonum and Stieda's process. However, whether these anatomical variations independently contribute to PAIS development remains unclear. This study aimed to investigate the independent association between posterior talar morphology and bony PAIS development using a propensity score-matched cohort design.

**Methods:**

This retrospective cohort study included 752 lateral ankle radiographs of patients aged 13–65 years between April 2018 and April 2021. Posterior talar morphology was classified as Group Ⅰ (os trigonum), Group II (isolated Stieda's process), Group III (large Stieda's process without separation from the talus), and Group IV (no posterior deformity). To control for confounding by age and sex, 1:4 propensity score matching was performed. The association between posterior talar morphology and bony PAIS was evaluated using conditional logistic regression adjusted for sports participation. Interrater reliability was assessed using Cohen's kappa coefficient.

**Results:**

Eighteen bony PAIS cases were matched to 72 controls. Adequate balance was achieved for age and sex (SMD<0.1). Interrater reliability was substantial (kappa = 0.633, p < 0.001). Os trigonum and isolated Stieda's process (Groups I and II) were significantly associated with bony PAIS (OR = 10.49, 95% CI 2.76–39.81, p < 0.001). Sports participation was not independently associated with bony PAIS (OR = 1.18, 95% CI 0.13–10.73, p = 0.884).

**Conclusions:**

Os trigonum and isolated Stieda's process were independently associated with an approximately 10-fold increased risk of bony PAIS, though these findings should be interpreted with caution given the limited number of cases. Patients with these morphological variants engaging in repetitive plantar flexion activities should be carefully monitored.

## 1 Introduction

Posterior ankle impingement syndrome (PAIS) represents a constellation of pathologies characterized by pain provoked by forced plantar flexion of the ankle, thereby leading to restriction in the normal range of motion of the joint.^1^ This condition is predominantly observed in athletes who require repetitive plantar flexion movements, such as the pointe position in classical ballet and kicking actions in soccer.^2^ Furthermore, PAIS has been reported to occur not only after these overuse events but also following direct trauma such as ankle sprain or fracture.^3^ Notably, ankle sprain may serve as a trigger for the onset of symptomatic PAIS in patients with previously asymptomatic os trigonum or Stieda's process, suggesting that trauma plays an important role in the transition from asymptomatic anatomical variation to clinically manifest PAIS.^4^ The pathogenesis of PAIS may result from both bone and soft tissue lesions and anatomic variations.^5^ Bony lesions include pathological os trigonum, Stieda's process (an elongated lateral tubercle to the talus), Shepherd's fracture (a fracture of the posterior process of the talus), osteophytes, osteochondral lesion, loose bodies, chondromatosis, and subtalar coalition.^2^, ^6^ Anatomically, at the ages of 11–13 years in boys and eight–10 years in girls, the secondary centers of ossification of the tubercles are visible posterior to the talus on radiographs.^7^ Generally, these ossification centers fuse with the talus within one year. However, these ossification centers fail to fuse in 7%–14% of individuals, referred to as os trigonum.^5^ Conversely, the further posterior elongation after fusion of the secondary ossification center is called the Stieda's process.^8^ The Stieda's process can sometimes lead to an acute fracture, called a Shepherd's fracture.^4^

Ribbans et al.^6^ reviewed 47 articles on PAIS and reported that 81% of 905 patients had bone impingement. Moreover, 827 patients (91.4%) were involved in sporting activities. However, reports regarding the presence of os trigonum on normal ankle radiographs widely varied.^9^ Therefore, anatomical features such as os trigonum or Stieda's process are not necessarily correlated with symptomatic PAIS, and the precise etiological factors of PAIS remain elusive. The aim of this study was to investigate the independent association between posterior talar morphology and bony PAIS development using a propensity score-matched cohort design.

## 2 Materials and methods

### 2.1 Patient selection

This study retrospectively reviewed 1727 ankles from 1241 patients who had lateral radiographs taken between April 2018 and April 2021 at a single institution. The inclusion criteria were as follows: (1) age between 13 years and 65 years; and (2) when bilateral radiographs were taken, the affected side was selected for analysis if the healthy side was used for comparison, or the right side was selected if bilateral symptoms existed. Cases were excluded if an evaluation by radiographs was not possible, such as after total ankle arthroplasty, joint immobilization, or incorrect lateral image. The study protocol was approved by the Ethics Committee of the Sapporo Medical University School of Medicine (authorization number 292-78). Given the retrospective nature of the study, the requirement for individual informed consent was waived, and an opt-out procedure was employed in accordance with institutional guidelines.

### 2.2 Radiographic and demographic evaluation

In the simple lateral radiographs, the morphology of the posterior talus was classified into four groups (Fig. 1). Group I was characterized by the presence of an os trigonum, which is an ossicle that does not completely fuse with the talus because of secondary ossification (Fig. 1a).^10^ Group II was characterized by the presence of an isolated Stieda's process (Fig. 1b), such as an os trigonum in fibrous continuity with the talus or a Shepherd's fracture, which is a fracture of the posterior process of the talus. Differentiating between Group I and Group II on radiographs is not always possible. Thus, Group I was characterized by an os trigonum with a round or oval morphology with distinct corticated margins, whereas Group II was characterized by irregular serrated margins between the talus and posterior segment; additionally, the fracture fragments may have smooth margins.^11^ Group III was characterized by a large Stieda's process without separation from the talus, presenting as a prominent posterior protrusion (Fig. 1c). Group IV was characterized by having no posterior deformity of the talus (Fig. 1d). Stieda's process was defined as the area where the posterior process of the talus protrudes posteriorly from the circle drawn along the ankle plane of the talus, based on the report by McAlister and Urooj^4^ (Fig. 1c). All images were independently classified by two examiners. Both examiners were blinded to the clinical diagnosis, including the presence or absence of bony PAIS, at the time of radiographic classification. Interrater reliability was assessed using Cohen's kappa coefficient. Age, sex, side on which the radiograph was taken (laterality), diagnosis, and level of sports participation were also recorded in the database.

**Fig. 1:**
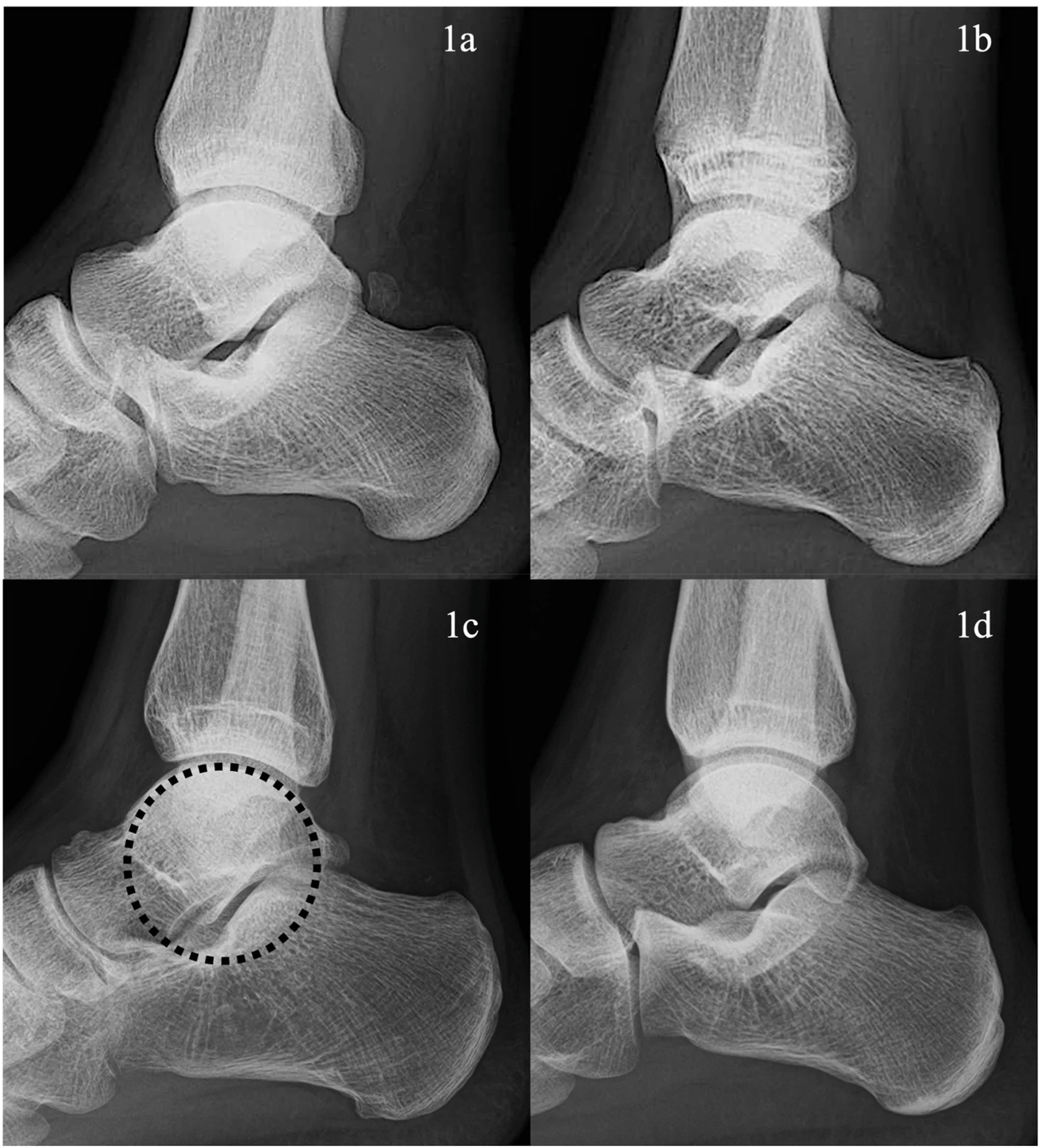
Classification of posterior talar morphology on lateral ankle radiograph. (a) Group I: os trigonum with round or oval morphology and distinct corticated margins. (b) Group II: isolated Stieda's process with irregular serrated margins at the talar junction. (c) Group III: large Stieda's process without separation, presenting as a prominent posterior protrusion. (d) Group IV: no posterior deformity.

Based on the diagnosis, patients were classified as having bony PAIS or not having bony PAIS. Bony PAIS was defined as the presence of pain in the hindfoot during plantar flexion force, posterior talar protrusion or free bodies on lateral radiographic images, and high signal in the posterior talus on T2-weighted magnetic resonance images. Magnetic resonance imaging (MRI)was performed only in patients with clinical suspicion of bony PAIS based on posterior hindfoot pain during plantar flexion and radiographic findings. As bony PAIS requires the presence of posterior hindfoot pain as a prerequisite for diagnosis, asymptomatic control patients were not considered to have bony PAIS regardless of radiographic morphology, and therefore did not undergo MRI. The level of sports participation was divided into the following two categories: daily and nondaily. The former category included patients who engaged in regular sports activity, whereas the latter category included patients who did not engage in regular sports activity. Sports participation was broadly categorized as daily versus non-daily, as detailed sport-type data were unavailable due to the retrospective nature of the study and inconsistent documentation in the medical records.

### 2.3 Statistical analysis

Data are presented as mean ± standard deviation for continuous variables and as number and percentage for categorical data. To control for potential confounding by age and sex, 1:4 propensity score matching was performed using the nearest-neighbor method. Matching balance was assessed using standardized mean differences (SMD), with SMD <0.1 indicating adequate balance between groups. Posterior talar morphology was dichotomized as os trigonum or isolated Stieda's process (Groups I and II) versus large Stieda's process or no posterior deformity (Groups III and IV), based on the presence or absence of a structurally separate osseous element in the posterior talus. Groups I and II were combined because both represent morphological variants in which the posterior talar process remains structurally non-fused or separated from the talus. These variants share a common anatomical mechanism that may predispose to repetitive entrapment during plantar flexion. The association between posterior talar morphology and bony PAIS development was evaluated using conditional logistic regression with sports participation included as a covariate, with matched sets defined as strata. Results are reported as odds ratios (ORs) with 95% confidence intervals (CI) and *p* values. Interrater reliability of the radiographic classification between two independent examiners was assessed using Cohen's kappa coefficient. Statistical significance was set at *p* < 0.05. All analyses were conducted using R version 4.4.0 (R Core Team 2024; R: A language and environment for statistical computing. R Foundation for Statistical Computing, Vienna, Austria. URL: https://www.R-project.org).

## 3 Results

Demographic and radiographic parameters are shown in Table 1. A total of 752 ankles from 752 patients were included in this study (Fig. 2). The cohort included 432 men and 320 women, with a mean age of 33.4 ± 17.8 years. A total of 417 feet were right-sided, and 335 feet were left-sided. In addition, 335 patients participated in daily sports activities, and 417 patients did not engage in regular sports activities. On simple lateral radiographs, posterior talar morphology was classified as follows: Group I in 27 (3.6%) feet, Group II in 147 (19.5%) feet, Group III in 370 (49.2%) feet, and Group IV in 208 (27.7%) feet. Eighteen patients (2.4%) were diagnosed with bony PAIS: three in Group I, 12 in Group II, and three in Group III. No patients in Group IV were diagnosed with bony PAIS.

**Table 1:** Demographic and radiographic parameters of all 752 patients.

	Group I (*n* = 27)	Group II (*n* = 147)	Group III (*n* = 370)	Group IV (*n* = 208)	All (*n* = 752)
Age, years	34.1 ± 2.0	29.2 ± 17.3	33.2 ± 17.2	36.5 ± 18.3	33.4 ± 17.8
Sex					
Male	15 (55.6)	87 (59.2)	232 (62.7)	98 (47.1)	432
Female	12 (44.4)	60 (40.8)	138 (37.3)	110 (52.9)	320
Laterality					
Right	16 (59.3)	83 (56.5)	203 (54.9)	115 (55.3)	417
Left	11 (40.7)	64 (43.5)	167 (45.1)	93 (44.7)	335
Diagnosis					
Bony PAIS	3 (11.1)	12 (8.2)	3 (0.8)	0 (0)	18
Other	24 (88.9)	135 (91.8)	367 (99.2)	208 (100)	734
Level of sports participation					
Daily	16 (59.3)	100 (68.0)	155 (41.9)	64 (30.8)	335
Nondaily	11 (40.7)	47 (32.0)	215 (58.1)	144 (69.2)	417

*Note:* Data are presented as the number, number (%), or mean ± standard deviation.

Abbreviation: PAIS, posterior ankle impingement syndrome.

**Fig. 2:**
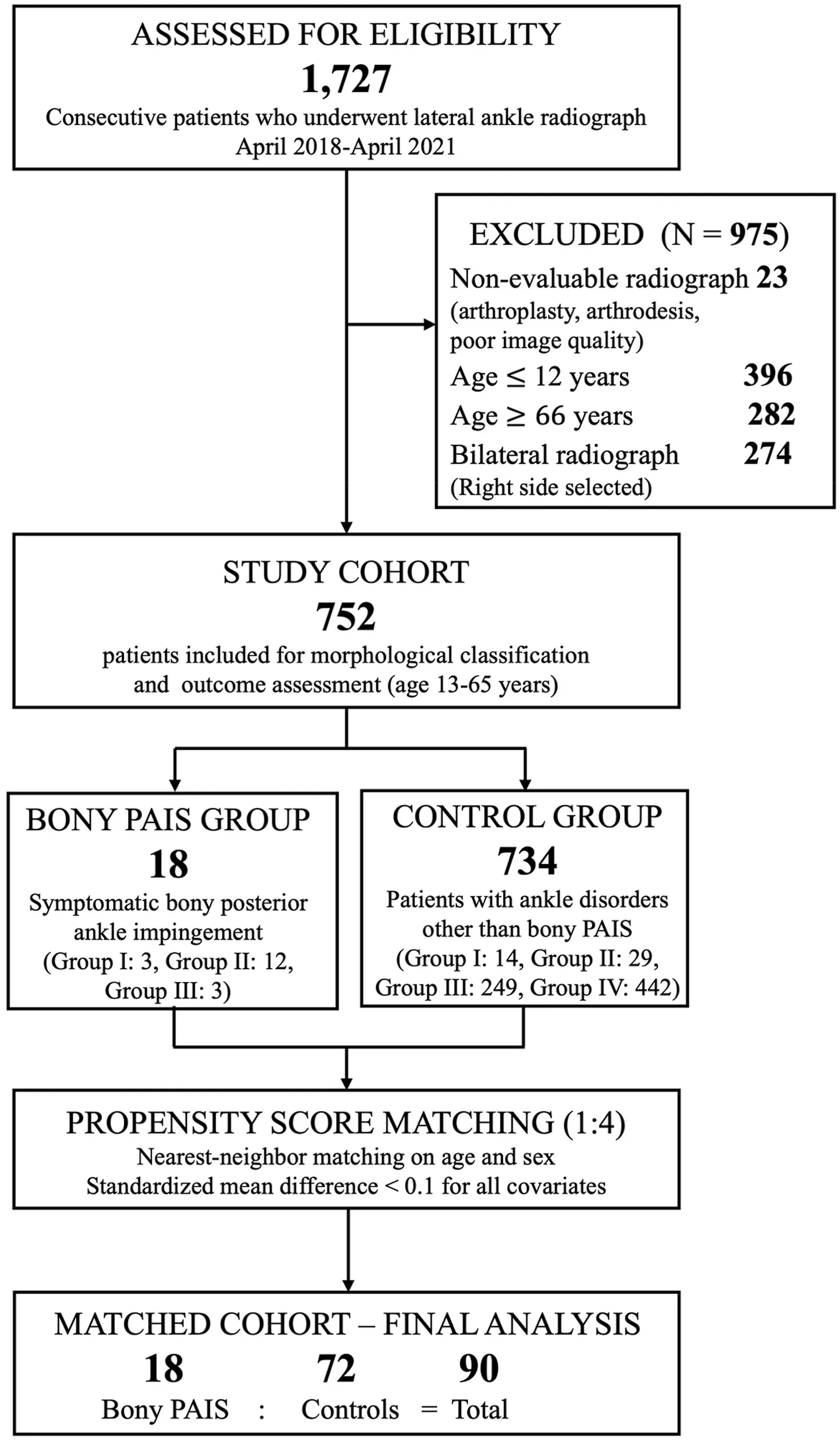
Flow diagram of patient selection. PSM, propensity score matching; PAIS, posterior ankle impingement syndrome.

After 1:4 propensity score matching based on age and sex, 18 bony PAIS cases were matched to 72 controls, yielding a total analytic sample of 90 patients. Adequate balance between groups was achieved for age (SMD = 0.001) and sex (SMD = 0.027). Sports participation showed an SMD of 0.296 between groups and was therefore included as a covariate in the subsequent regression model. Background characteristics of the matched cohort are shown in Table 2.

**Table 2:** Background characteristics of the matched cohort.

	Control (n = 72)	Bony PAIS (n = 18)	*p* value	SMD
Mean age, years	21.01 ± 10.89	21.00 ± 11.00	0.996	0.001
Sex (male/female)	39/33	10/8	0.917	0.027
Level of sports participation (daily/nondaily)	51/21	15/3	0.289	0.296

*Note:* The matched cohort was derived from 1:4 propensity score matching based on age and sex. Standardized mean differences (SMD) < 0.1 indicate adequate balance between groups. *p* values were calculated using conditional logistic regression within matched sets.

Interrater reliability of the radiographic classification of posterior talar morphology between two independent examiners was substantial, with a Cohen's kappa coefficient of 0.633 (p < 0.001).

The results of the conditional logistic regression are shown in Table 3 and Fig. 3. The presence of os trigonum or isolated Stieda's process (Groups I and II) was significantly associated with bony PAIS development after adjusting for sports participation (OR = 10.49, 95% CI 2.76–39.81, p < 0.001). Sports participation was not independently associated with bony PAIS development (OR = 1.18, 95% CI 0.13–10.73, p = 0.884).

**Table 3:** Conditional logistic regression analysis for bony PAIS development.

	OR	95% CI	*p* value
Posterior talar morphology (Groups I and II vs. III and IV)	10.49	2.76-39.81	<0.001
Sports participation (daily vs. nondaily)	1.18	0.13-10.73	0.884

*Note:* Abbreviation: CI, confidence interval; OR, odds ratio; PAIS, posterior ankle impingement syndrome.

**Fig. 3:**
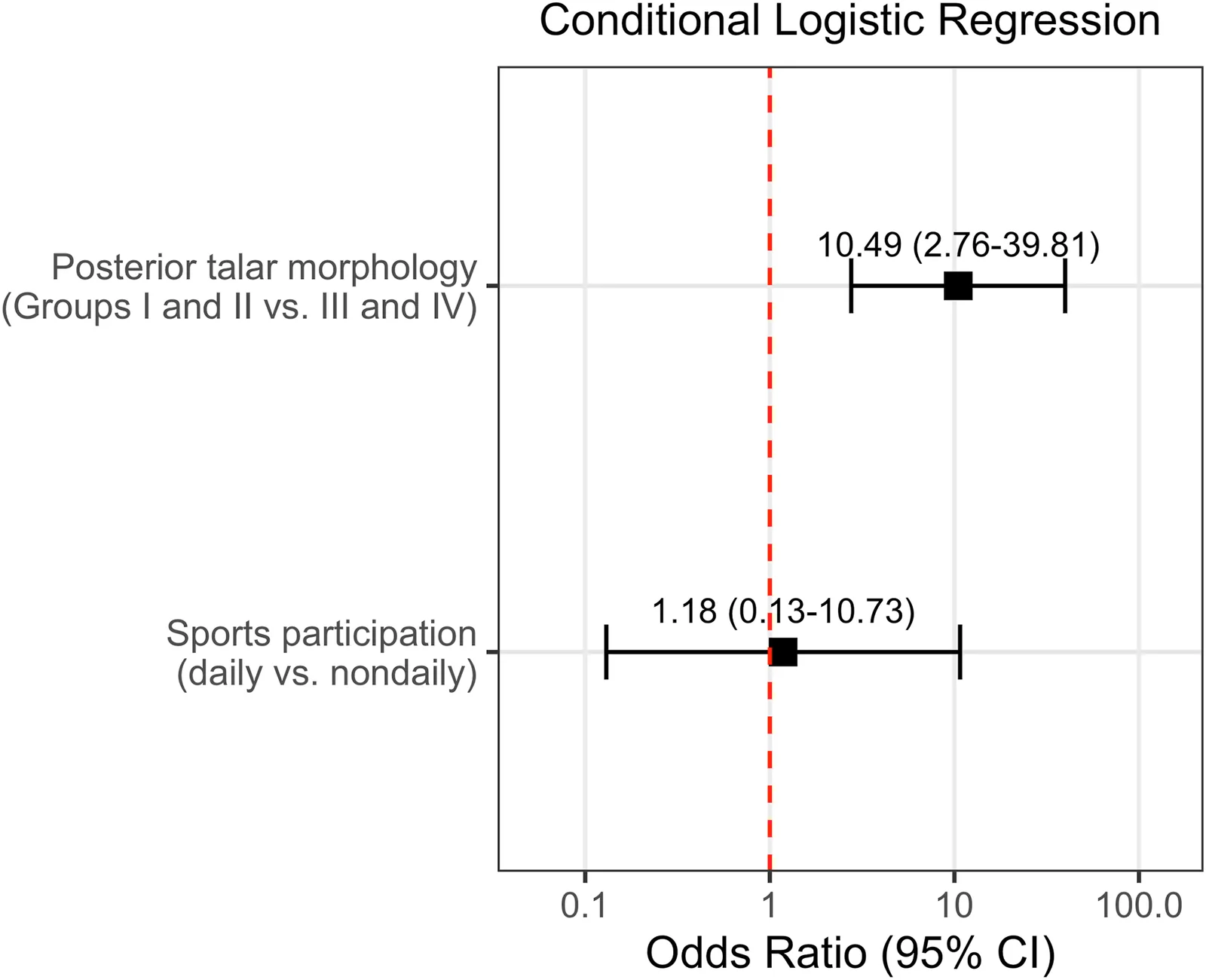
Forest plot of conditional logistic regression analysis for bony posterior ankle impingement syndrome (PAIS) development in the propensity score-matched cohort (18 bony PAIS, 72 controls). Squares represent odds ratios and horizontal lines represent 95% confidence intervals. The dashed vertical line indicates an odds ratio of 1.0. PAIS, posterior ankle impingement syndrome.

## 4 Discussion

This study investigated the factors affecting the development of bony PAIS based on simple lateral radiographs using a propensity score-matched cohort design. Our most important finding was that the presence of os trigonum or isolated Stieda's process was independently associated with an approximately 10-fold increased risk of bony PAIS development after controlling for age, sex, and sports participation. In addition, our study findings indicate that many patients have asymptomatic os trigonum and Stieda's process.

Although some studies^8^, ^12^, ^13^ suggest that the prevalence of PAIS ranges from 1.7% to 30%, the true incidence of PAIS is unknown. The uncertainty regarding the true incidence of PAIS is because it can be misdiagnosed, owing to the low index of suspicion for the condition and the fact that symptoms often improve with rest alone.^5^ Furthermore, some studies^14^, ^15^, ^16^, ^17^ have reported on the prevalence of os trigonum. One report^17^ showed that the prevalence of radiologically detectable os trigonum ranges from 3% to 13% in the general population. Another study^16^ indicated that the incidence of os trigonum ranged from 14% to 25% on normal radiographs of the ankles and feet. Burman and Lapidus^14^ reported a 50% prevalence of os trigonum in all feet. Fu et al.^15^ investigated the prevalence of os trigonum in the normal ankle of Chinese individuals by using computed tomography and found that 27.2% of individuals had os trigonum. Among these individuals, 1.9% had type I (i.e., a single piece of bone not connected to the talus), 10.5% had type II (i.e., os trigonum connected to the posterior talar process by hyaline cartilage), and 14.7% had type III (i.e., Stieda's process). In our study, 3.2% of patients had asymptomatic os trigonum and 18.0% had isolated Stieda's process, comparable to findings in previous radiographic reports,^14^, ^16^, ^17^ although each type was more frequent than that reported in a computed tomography-based study.^15^ Therefore, radiographic assessment can be considered equivalent to computed tomography assessment and is effective for evaluating os trigonum. The results of the present study also indicated that os trigonum and isolated Stieda's process are more likely to cause PAIS and that approximately 23% of patients had these morphological variants, highlighting the importance of careful monitoring in this population.

One mechanism underlying PAIS has been described as a ‘nut in a nutcracker’.^18^ After the Stieda's process forms, repeated excessive ankle plantar flexion can fracture the Stieda's process and damage fibrocartilaginous connective tissue. This leads to fibrosis, scarring, and hardening of the site, resulting in compression between the calcaneus and the tibia. This repetitive compression causes stress on the joint capsule and soft tissue, leading to inflammation. The current study identified os trigonum and isolated Stieda's process as independent risk factors for PAIS development, whereas large Stieda's process without separation (Group III) was not independently associated with PAIS. The dichotomization of posterior talar morphology into Groups I and II versus III and IV was supported by the results, as no patients in Group IV developed bony PAIS. These findings suggest that the structural independence of the posterior talar process, rather than its size alone, may be a critical determinant of bony PAIS development. However, the posterior talar process may also separate because of trauma, as in a Shepherd's fracture. Thus, attention must be paid to significantly protruding Stieda's processes. The risk factors for such fractures remain unclear, necessitating further research.

Sports participation was included as a covariate in the regression model rather than incorporated into the propensity score matching procedure, because the limited sample size of 18 bony PAIS cases restricted the number of matching variables. Incorporating additional covariates into the PSM would have reduced the number of matched pairs and further compromised statistical power. The finding that sports participation was not independently associated with bony PAIS in the present study (OR = 1.18, p = 0.884) should be interpreted with caution. Sports participation was broadly dichotomized as daily versus non-daily; therefore, this result does not imply that physical activity cannot trigger symptomatic impingement. Rather, it suggests that the simple binary categorization used in this study may have been insufficient to capture the nuanced relationship between activity level and PAIS development. Keceli reported that os trigonum size was the most important factor in PAIS development in pediatric patients; the os trigonum was significantly larger in symptomatic than in asymptomatic individuals (9.0 mm vs. 6.7 mm, p = 0.04), whereas sex, age, and skeletal maturity showed no significant association with PAIS.^19^ Abramowitz et al. similarly demonstrated that, although athletes tended to have better surgical outcomes than non-athletes with os trigonum syndrome, multivariate regression analysis identified symptom duration—not sports participation—as the only independent predictor of postoperative outcome.^20^ Furthermore, D'Hooghe et al. demonstrated that even among professional athletes with os trigonum syndrome, chronic lateral ankle instability—rather than sports participation alone—was required to predict surgical intervention, suggesting that additional anatomical factors modulate symptom expression beyond activity level.^3^ Collectively, these findings suggest that posterior talar morphology may represent an underlying anatomical predisposition for bony PAIS, while the role of activity level warrants further investigation with more detailed sport-specific data.

This study has some limitations. First, as this was a retrospective study based on archived medical records, documentation of physical examination findings may have been incomplete for some patients. Although MRI was performed in all 18 patients in the bony PAIS group, it was not obtained in all patients in the control group, as asymptomatic patients were not considered to have bony PAIS regardless of radiographic morphology. This information bias inherent to retrospective chart review may have affected case ascertainment. Second, it was conducted at a single facility, potentially limiting the generalizability of the findings to broader populations with different demographics or clinical practices. Third, although interrater reliability of the radiographic classification was substantial (Cohen's kappa = 0.633), the classification relied solely on radiographic data. Advanced imaging modalities such as computed tomography or magnetic resonance imaging may provide more precise morphological assessment. Furthermore, differentiating Group I (os trigonum) and Group II (isolated Stieda's process) on plain lateral radiographs is inherently challenging due to subjective interpretation during image reading, which may have introduced misclassification between these two groups. Fourth, daily sports participation was broadly defined as regular sports activity without stratifying by type or intensity, which could influence PAIS development differently. For many patients, the type of sport was not specified, owing to insufficient detail in patient data and the unclear association between the injury and specific sports activities. Future studies should stratify sports by type and intensity and conduct statistical analyses to elucidate the risk factors associated with the development of PAIS. Fifth, prior ankle sprain and chronic lateral ankle instability, which are recognized triggers for posterior ankle impingement syndrome, were not collected in this study. Accordingly, residual confounding from these factors cannot be excluded, and their potential contribution to bony PAIS development warrants investigation in future studies. Finally, only 18 cases of bony PAIS were identified, limiting the statistical power of the analysis. Although propensity score matching effectively controlled for confounding by age and sex, the small number of cases may have led to result fluctuation and wide confidence intervals for risk estimates, limiting the generalizability of the findings. The conclusions should therefore not be overstated, and future multicenter studies with larger sample sizes are warranted to confirm these findings.

In conclusion, os trigonum and isolated Stieda's process were independently associated with an approximately 10-fold increased risk of bony PAIS development after controlling for age, sex, and sports participation through propensity score matching, although these findings should be interpreted cautiously given the small sample size and the retrospective study design. Patients with these morphological variants who engage in repetitive plantar flexion activities may be carefully monitored for PAIS development. Further multicenter studies are needed to confirm these findings and elucidate additional risk factors including the role of prior ankle trauma.

## Authors’ contributions

YM and TM carried out the data analysis. YM designed the study plan. YM conducted this study. TK, ME, and KW collected the data and assisted the experiment. AT organized this study. All authors read and approved the final statistical analysis.

## Ethics declaration

Written informed consent to take part in the study and to publish the article has been obtained from all participants or their legal representatives. The privacy rights of participants have been observed.

This study was performed in compliance with relevant laws, regulatory frameworks and guidelines where the research took place.This study was approved by the Sapporo Medical University School of Medicine.(Approval No. 292-78)

## IRB information

This study was approved by the Ethics Committee of the Sapporo Medical University School of Medicine (authorization number 292-78).

## Declaration of generative AI and AI-assisted technologies in the writing process

During the preparation of this work, the authors used Claude (Anthropic) to assist with language refinement and statistical analysis support. All outputs were reviewed and edited by the authors, who take full responsibility for the content of the publication.

## Funding

This research did not receive any specific grant from funding agencies in the public, commercial, or not-for-profit sectors.

## Declaration of competing interest

The authors declare that they have no known competing financial interests or personal relationships that could have appeared to influence the work reported in this paper.
